# P03.17. A fellowship program for CAM providers in evidence informed geriatrics

**DOI:** 10.1186/1472-6882-12-S1-P270

**Published:** 2012-06-12

**Authors:** C Vihstadt, K Westrom, R Evans

**Affiliations:** 1 Northwestern Health Sciences University, Bloomington, USA

## Purpose

Elderly residents in long term care (LTC) settings experience a myriad of health complaints that may be amenable to complementary and alternative medicine (CAM) therapies. However, specialized training addressing the unique features of geriatric health care, particularly in LTC settings, will be required if CAM professionals are to play a meaningful role. The purpose of this presentation is to describe an evidence informed geriatric fellowship program at a CAM institution which educates acupuncture, chiropractic and massage therapy practitioners.

## Methods

Six core competencies and 20 learning objectives were identified for preparing the CAM practitioners to deliver care in LTC. These were addressed throughout the fellowship using a variety of training approaches such as weekly seminar-style meetings, facilitated discussions, directed readings, clincial demonstration and practice, online learning programs, and individual meetings. The core competencies focused on the skills needed to provide care safely within LTC and to a frail population. Training concentrated on preparing pracitioners to find and utilize the best available research for clinical decision making and how to effectively communicate with other health workers, patients and family members.

## Results

Six fellows participated in the fellowship and provided services in LTC. Five fellows completed the program. Of these, all have utilized the experience to further their careers in education and research at CAM institutions. Individuals have gone on to teach evidence informed practice, specialized geriatrics techniques, and supervise a student intership in geriatrics in LTC. One fellow serves as Director of Consultative Services in a skilled nursing facility; another is enrolled in a Clinical Research Fellowship with a career focus on musculoskeletal conditions and CAM for seniors.

## Conclusion

A fellowship program designed to develop the skills CAM practitioners require to work with senior and frail populations can be effective in furthering the role of CAM in LTC settings and research.

